# Effects of melatonin supplementation on oxidative stress, and inflammatory biomarkers in diabetic patients with chronic kidney disease: a double-blind, randomized controlled trial

**DOI:** 10.1186/s40795-025-01026-0

**Published:** 2025-02-08

**Authors:** Sara Sadeghi, Amirahmad Nassiri, Monir Sadat Hakemi, Fatemeh Hosseini, Fatemeh Pourrezagholie, Fatemeh Naeini, Aylar Nadiri Niri, Hossein Imani, Hamed Mohammadi

**Affiliations:** 1 Department of Clinical Nutrition, School of Nutritional Sciences and Dietetics, Tehran University of Medical Sciences, Tehran, Iran; 2 Department of Nephrology, Imam Hossein Hospital, Shahid Beheshti University of Medical Sciences, Tehran, Iran; 3 Department of Nephrology, Shariati Hospital, Tehran University of Medical Sciences, Tehran, Iran; 4 Department of Nephrology, Labbafinezhad Hospital, Shahid Beheshti University of Medical Sciences, Tehran, Iran; 5 Cell and Molecular Biology Department, University of Tehran, Tehran, Iran

**Keywords:** Chronic kidney disease, Melatonin, Oxidative stress, Inflammation

## Abstract

**Background and objectives:**

Chronic kidney disease (CKD) is a progressive illness linked to higher rates of morbidity and death. One of the main causes of CKD is diabetes mellitus (DM), and oxidative stress is essential to the disease's development. It has been demonstrated that the natural antioxidant melatonin reduces inflammation and oxidative damage in renal tissues. Given the lack of robust evidence, this double-blind clinical trial sought to investigate the effects of melatonin supplementation on oxidative stress and inflammatory markers in diabetic CKD patients.

**Materials and methods:**

This trial included 41 diabetic patients with CKD (stages 3–4) from Shariati Hospital, Tehran, Iran. For ten weeks, participants were randomized to receive either a placebo or 5 mg of melatonin twice a day. Baseline characteristics, dietary intake, physical activity, and anthropometric measurements were recorded. Oxidative stress (TAC, TOS, MDA) and inflammatory markers (IL-6, hs-CRP) were measured before and after the intervention. Statistical analysis was performed using SPSS, with significance set at p < 0.05.

**Results:**

The 10-week trial was completed by 41 participants in total, and no adverse effects were noted. Dietary intake, physical activity, and anthropometric parameters did not significantly differ between the melatonin and control groups in baseline characteristics. Melatonin supplementation decreased oxidative stress and inflammatory biomarkers, including hs-CRP, MDA, TOS, and IL-6. However, these changes were not statistically significant.

**Conclusion:**

Our study showed that melatonin supplementation did not significantly affect oxidative stress or inflammatory markers, including TAC, TOS, MDA, IL-6, and hs-CRP, in diabetic patients with CKD. Despite a decrement in TOS, MDA, IL-6, and hs-CRP levels after 10 weeks, this was not statistically significant. Further studies with larger sample sizes, greater dosages, and longer follow-up periods are recommended.

## Introduction

Chronic kidney disease (CKD) is one of the most prevalent medical conditions in the twenty-first century that arises from the progressive and irreversible destruction of renal nephrons quantity and function. Diagnosing CKD involves lowering the glomerular filtration rate (GFR) to less than 60 ml /min/ 1.73 m^2^ or detecting kidney damage, or both, for a minimum of three months [1, 2]. The kidney transplant or dialysis is the last course of treatment for individuals whose GFR falls to less than 15 ml/min/ 1.73 m^2^, indicating the end stage of the illness. We refer to this state as ESRD [1]. The likelihood of an early death in CKD patients is, however, far greater than progressing the condition to its terminal stage and developing ESRD [3, 4]. Initial studies show that CKD is projected to be the fifth most common cause of years of life lost by 2040 [5]. CKD is mostly associated with four major risk factors: polycystic kidney disease (PKD), glomerulonephritis, diabetes mellitus (DM), and hypertension (HTN) [2, 6, 7]. Thus, the adult population with DM and HTN is more likely to develop CKD eventually [2, 3]. Research indicates that diabetics, as the most prevalent cause of ESRD, have a 20–40% risk of developing CKD [2, 8]. Diabetic kidney disease (DKD) is when a diabetic patient has consistent proteinuria, albuminuria, and/or a GFR below 60 ml/min/1.73 m2 [9]. Due to the high prevalence of DKD, which increases disease-related morbidity and mortality, present approaches focus on prevention and early treatment [10].

Prior research highlights the importance of inflammation and oxidative stress to the onset of CKD [11]. Inflammatory processes are capable of generating increased levels of reactive oxygen species (ROS) and triggering the activation of the NF-kB pathway. However, they may also increase the number of apoptotic proteins by releasing pro-inflammatory cytokines like TNF-alpha [12]. Furthermore, NOX enzyme as an oxidative enzyme induces the degeneration of renal nephrons and sclerosis of mesangial cells by persistently activating the RAS system, which raises ROS production and disturbs sodium homeostasis [13–15].

Therefore, because of the substantial role of oxidative stress in CKD development, treating these patients with antioxidants might be helpful [16]. Melatonin, also called N-acetyl 5-methoxytryptamine, is a naturally produced indoleamine found in the body, such as in the pineal gland and kidneys [17]. Furthermore, previous research indicated that the renal disease stage correlates with the level of melatonin production rhythm disruption and function decline [18]. Melatonin has multiple mechanisms through which it plays its antioxidant role. These mechanisms include direct elimination of free radicals, regulation of apoptosis and autophagy [19], improvement of antioxidant enzyme function [20], reduction of lipid peroxidation and renal fibrosis [21], and prevention of excessive immune system cell penetration into kidney tissue [22].

Due to melatonin's antioxidant properties and given the lack of robust evidence in this regard, this double-blind clinical trial was carried out to investigate the effect of melatonin supplementation on oxidative stress and inflammatory markers in these patients.

## Methodology

### Study design and participants

Diabetic patients with CKD in the stages before dialysis (stages 3–4) referred to the Shariati Hospital, associated with Tehran University of Medical Sciences, Tehran, Iran, participated in this double-blind, placebo-controlled, parallel randomized clinical trial (allocation ratio: 1:1). A nephrologist utilized laboratory findings to confirm the presence of CKD. Every patient was made aware of the purpose and methods of the study, and their informed written permission was acquired. This research was registered in the Iranian registry of clinical trials (IRCT ID: IRCT20170202032367N9, Registration date: 2023–08–11, Trial Id: 70709). Our study adheres to CONSORT guidelines.

The following were the defined inclusion criteria: Participants must be between the ages of 25 and 65, have a body mass index (BMI) of more than 20 and less than 30 kg/m^2^, and be willing to participate in the study. Participants must also have been diagnosed with DM and CKD in the stages before dialysis (stages 3–4) by a specialist based on laboratory findings. Patients were not be accepted into the study if they: (1) have glomerulonephritis or autoimmune kidney disease; (2) have blood pressure (BP) greater than 160/100 mmHg; (3) are pregnant, lactating, or intend to become pregnant within the next six months; (4) have inflammatory, infectious, thyroid gland, or thrombocytopenia disorders; (5) are receiving enteral and parenteral nutritional support; (6) have a history of taking antioxidant and omega-3 supplements (zinc, selenium, vitamin E, vitamin C, vitamin B6, and beta-carotene separately) from three months before the study; (7) are taking warfarin, glucocorticoids in doses greater than 5 mg, antibiotics, fluvoxamine, non-steroidal anti-inflammatory drugs (NSAIDs); (8) are smokers; and (9) hold career paths that require night shifts. The following were the exclusion criteria: Those who chose not to continue taking supplements, those who became pregnant during the trial, those who did not take more than 10% of supplements at each follow-up, and those who went into dialysis or kidney transplant stage were the groups of people identified. The patients received instructions on taking their supplements and were contacted once a week to ensure they were taking the supplements as prescribed as part of the ongoing research. The remaining number of capsules was counted at the halfway point and conclusion of the study to assess if the intervention was being carried out; a percentage of less than 90% was deemed to indicate low compliance. The Medical Ethics Committee of Tehran University of Medical Sciences, Tehran, Iran (IR) granted this study's ethical approval.TUMS.SHARIATI.REC.1402.072(. Informed consent was obtained from all participants. All identifying information was removed from the data files and replaced with unique study IDs to ensure anonymity. Data were stored on secure, password-protected servers accessible only to authorized research personnel.

A total of 41 eligible diabetic patients with CKD were randomized into two groups: the intervention group (*n* = 20) and the control group (*n* = 21). Each group was given melatonin (5 mg) and placebo capsules that looked the same, two times daily for 10 weeks. The Karen Company, Yazd, Iran, provided both the melatonin and placebo capsules. Melatonin and placebo capsules were indistinguishable in terms of weight, size, shape, flavor, color, smell, and lot number, with the latter containing starch. Participants were encouraged to continue their usual eating habits and exercise routines during the intervention. There will be no special criteria for discontinuing or modifying allocated interventions. Melatonin or Placebo will not require alteration to usual care pathways (including the use of any medication) and these will continue for both trial arms.

### Justification of sample size, randomization, and blinding

Based on the type I error of 5% (α = 0.05) and type II error of 20% (β = 0.20, power = 80%) and total antioxidant capacity (TAC) as the main variable [23], the estimated needed sample size of 22 participants was computed for each group. We performed the sample size calculations using G*Power version 3.1 [24], which is a widely recognized statistical power analysis software that assists in determining appropriate sample sizes for various study designs. The sample size was increased to 24 individuals per group, taking into account a 10% dropout rate.

We employed stratified block randomization after participant recruitment, where individuals were divided into distinct blocks according to their sex (male/female) and age (under 30 and over 30). An age- and sex-matched individual was assigned to a different block for every patient in a certain block. Two individuals from the same neighborhood were later assigned randomly to either the intervention or control groups. We employed a 1:1 allocation ratio to ensure equal representation in both groups. Using a randomly generated sequence created by a computer, a third person who was not aware of the study's purpose assigned participants at random (sequentially numbered).

All patients, researchers, nephrologists, and laboratory staff were blinded to the intervention.

### Outcomes

Total antioxidant capacity (TAC), total oxidative stress (TOS), malondialdehyde (MDA), as oxidative stress indicators, and inflammatory markers including interleukin-6 (IL-6) and highly sensitive C reactive protein (hs-CRP), are the key outcomes of the current study.

#### Assessment of dietary intake and physical activity

Participants were asked to provide three 24-h dietary recalls at the start, week 5, and conclusion of the study. They were also instructed to fill out the approved brief version of the International Physical Activity Questionnaire (IPAQ-SF). The dietary data was assessed using the Nutritionist IV software program. The IPAQ questionnaire reported the amount of metabolic equivalent work per week (MET-min/wk) of physical activity [25]. We analyzed the IPAQ-SF data using the IPAQ scoring protocol [26], which is a standardized method for converting self-reported physical activity data into MET-min/week scores. The IPAQ scoring protocol categorizes physical activity into three domains: walking, moderate-intensity physical activity, and vigorous-intensity physical activity. The MET values for each domain are as follows: walking (3.3 METs), moderate-intensity physical activity (4–5.5 METs), and vigorous-intensity physical activity (6–8 METs). We calculated the total MET-min/week score by multiplying the duration and frequency of each activity type by its corresponding MET value and summing the results.

#### Anthropometric measurements

The trial's beginning and ending involved the measurement of anthropometric variables, such as height, weight, and waist circumference (WC). While the participants were standing, wearing light clothes, and without shoes, their weight and height were recorded using the Seca scale (Seca Co., Hamburg, Germany) and stadiometer (Seca) to the closest 0.1 kg and 0.1 cm, respectively. The weight/height^2^ (kg/m^2^) formula was used for calculating BMI. Between the iliac crest and the lowest rib, WC was measured using a non-elastic tape**.**

#### Biochemical measurements

After a 12-h overnight fast, 10 mL of venous blood was collected and serum samples were extracted both before and after the intervention using a centrifuge set at 3500 rpm for ten minutes. Serum samples were kept at -80 °C for further examination. The commercial enzyme-linked immunosorbent assay (ELISA) kits were used to measure blood concentrations of oxidative stress biomarkers and inflammatory factors (TAC, TOS, MDA, IL-6, and hs-CRP). The levels of TAC, TOS, MDA were assessed using the Navand-Salamat ELISA kits. The TOS and MDA kits demonstrated a sensitivity of 0.5 µmol/L and a specificity of greater than 95%. The sensitivity of the TAC kit was 0.1 mmol/L and its specificity was more than 92%. Serum levels of IL-6 were measured using the ELISA kit from Kit-Iran company. According to the manufacturer’s information, the kit has a sensitivity of 0.3 pg/mL and exhibits minimal cross-reactivity with other cytokines. Specifically, the kit demonstrates less than 1% cross-reactivity with TNF-α, and IL-1β. Hs-CRP levels were quantified using an ELISA kit provided by Kit-Iran. The manufacturer reports a sensitivity of 0.02 µg/mL for this assay. This kit is specifically designed for measuring hs-CRP with minimal interference from other C-reactive protein forms. The assay procedure adhered to the guidelines provided by the kit manufacturer.

### Statistical analysis

For all analyses, SPSS software version 25 (SPSS Inc., Chicago, IL, USA) was used. The Kolmogorov–Smirnov test was used to determine if the data had a normal distribution. Mean ± standard deviation (SD) was used to represent quantitative variables having a normal distribution, whereas the 25–75th interquartile range was used to report data with a non-normal distribution. The frequency and percentage of the qualitative data were also presented. To evaluate the variations within each group, the Paired-Samples t-test or its non-parametric counterpart, the Wilcoxon Signed-Rank test, was employed. The Chi-Square and Mann–Whitney U tests were used for nominal variables and the Independent-Samples t-test or Mann–Whitney U test was used for quantitative variables comparing between the research groups. Additionally, the generalized linear model was employed to adjust the data for any cofounders. Statistical significance was established as having a p-value lower than 0.05. In cases of missing data; statistical analysis was performed using the intention-to-treat (ITT) method.

## Results

**Fig. 1:**
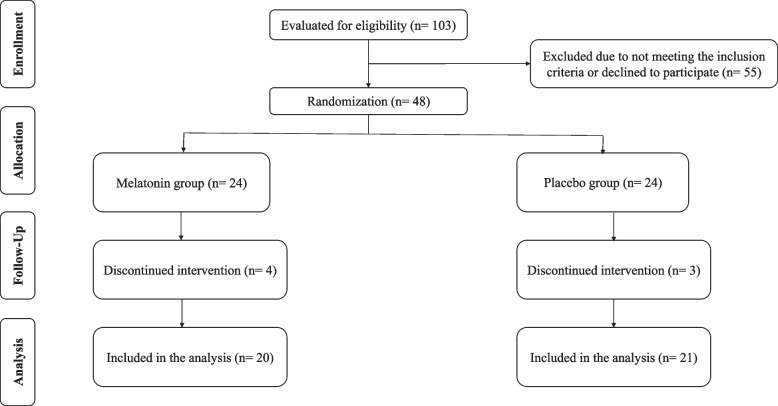
Study flowchart

As shown in Fig. 1, 48 subjects met the criteria and enrolled in the study. Seven of the patients were lost to follow-up, finally, 41 subjects (21 individuals in the control group and 20 individuals in the melatonin group) completed the 10-week trial. Participants tolerated the melatonin supplement and no side effects were reported. participant followed the supplementation protocol exactly, and no adverse effects were recorded.

### Characteristics of the participants

Table 1 illustrates the individuals' baseline characteristics. Age, weight, sex, education, marital status, and the usage of medications such as insulin, metformin, and statins were not significantly different between the melatonin and control groups at baseline (*p* > 0.05). The mean age of the study participants was 64 years (SD 12) in the intervention group and 65 years (SD 12) in the control group (Table 1).

**Table 1:** Baseline characteristics of the study participants

Variables		Groups		*P*-Value ^a^
		Melatonin (*n* = 20)	Control (*n* = 21)	
Age (years)		64 ± 12	65 ± 12	0.84†
Weight (kg)		78.9 ± 9	82.1 ± 12.8	0.36†
Sex	Female	3 (15)	4 (19)	0.73*
	Male	17 (85)	17 (81)	
Marital status	single	2 (10)	1 (4.8)	0.52*
	married	18 (90)	20 (95.2)	
Education	Diploma and lower	14 (70)	16 (77)	0.78*
	Bachler and higher	6 (30)	5 (23)	
Statin	Yes	19 (95)	18 (85.7)	0.31*
	No	1 (5)	3 (14.3)	
Metformin	Yes	1 (5)	6 (28.6)	0.4*
	No	19 (95)	15 (71.4)	
Insulin	Yes	9 (45)	14 (66.7)	0.16*
	No	11 (55)	7 (33.3)	

Data are presented as mean ± SD for quantitative and frequency (%) for qualitative variables

^a^ based on †independent sample t test, and * chi-square

### Dietary intake and physical activity

Table 2 shows the dietary intake and physical activity levels of the study participants both before and after the 10-week intervention. At baseline, there were no noticeable differences in the two groups' calorie intake, macronutrient distribution, or levels of physical activity (*p* > 0.05). Moreover, there was no significant change in the mentioned factors across the duration of the intervention (*p* > 0.05) (Table 2).

**Table 2:** Daily dietary intake and physical activity of the study subjects at baseline and after the 10-week intervention

Variables	Time	Groups		*P*-Value ^a^
		Melatonin (*n* = 20)	Control (*n* = 21)	
**Energy (kcal/d)**	**Before**	**1537.8 ± 538.6**	**1441.8 ± 392.5**	0.52^**†**^
	**After**	**1510.1 ± 436.3**	**1341.3 ± 320.7**	0.16^**†**^
	**Change**	**-27.7 ± 307.34**	**-100.4 ± 233.2**	0.4^**†**^
**Carbohydrate (g/d)**	**Before**	**214.3 ± 77.4**	**201.1 ± 68.4**	0.56^**†**^
	**After**	**211.03 ± 68.7**	**194.4 ± 62.7**	0.42^**†**^
	**Change**	**-3.35 ± 49.1**	**-6.63 ± 43.1**	0.82^**†**^
**Protein (gr/d)**	**Before**	**56.9 (41.2–64.35)**	**52.06 (35.9–71.5)**	0.54^**‡**^
	**After**	**54.72 (40.9–68.8)**	**49.1 (42.5–54.5)**	0.67^**‡**^
	**Change**	**-2.24 (-5, 6.04)**	**-2.9 (-10.16–8.4)**	1.0^**‡**^
**Fat (g/d)**	**Before**	**52.5 ± 21.4**	**50.2 ± 17.2**	0.71^**†**^
	**After**	**52.5 ± 14.3**	**43.5 ± 12.5**	0.39^**†**^
	**Change**	**0.05 ± 16.35**	**-6.7 ± 12.6**	0.15^**†**^
Physical activity	Before	693 (445.5–1386)	594 (445.5–1386)	0.86^**‡**^
	After	717.8 (231–1386)	990 (462–1386)	0.48^**‡**^
	Change	24.7 (0–247.5)	0 (-66, 327)	0.89^**‡**^

Data are presented as mean ± standard deviation or Median (IQR)

^a^ based on ^**†**^independent sample t-test, and ^**‡**^ Mann–Whitney U-test

### Anthropometric parameters

Table 3 provides information on anthropometric parameters such as weight, BMI, and waist circumference (WC). Following the 10-week intervention, there were no significant differences in weight, BMI, or WC in the melatonin group compared to the control groups (*p* > 0.05) (Table 3).

**Table 3:** Anthropometric parameters of the study subjects at baseline and after the 10-week intervention

Parameter	Time	Groups		Mean difference	95% CI		*P* ^a^
		Melatonin (*n* = 20)	Control (*n* = 21)		Lower	Upper	
Weight	Before	78.9 ± 9	82.1 ± 12.8	-3.1	-10.1	3.8	0.36^**†**^
	After	78.1 ± 9.4	82.0 ± 12.3	-0.8	-2.6	0.89	0.25 ^**ƪ**^
	Change	-0.77 ± 1.99	-0.03 ± 3.38	-3.1	-10.1	3.8	0.39^**†**^
BMI	Before	29.2 ± 3.54	29.9 ± 4.54	-0.7	-3.3	1.8	0.56^**†**^
	After	28.9 ± 3.63	29.9 ± 4.59	-0.3	-0.9	0.35	0.34^**ƪ**^
	Change	-0.29 ± 0.73	0.2 ± 1.29	-0.3	-0.9	0.35	0.34^**†**^
WC	Before	106 (103, 110)	112 (106, 113)	-3.2	-9/05	2.65	0.1^**‡**^
	After	107 (105, 110)	110 (107, 114)	-0.88	-2.6	0.89	0.31 ^**ƪ**^
	Change	-1 (-1.5, 2)	-0.5 (−2, 2)	-0.75	-1.87	1.72	0.9^**‡**^

Data are presented as mean ± standard deviation

^a^ based on ^**†**^independent sample t-test, ^**‡**^ Mann–Whitney U-Test, and ^**ƪ**^ general linear model

### Biochemical parameters

Table 4 displays the participants' pre- and post-intervention biochemical data. Serum levels of hs-CRP, TOS, IL-6, TAC, and MDA did not significantly change between the melatonin and control groups at baseline (*p* > 0.05). After melatonin supplementation, hs-CRP, MDA, TOS, and IL-6 levels decreased by 29%, 2%, 1%, and less than 1% respectively. However, these changes were not statistically significant (*p* > 0.05). Regarding changes in serum levels of IL-6, there was a significant difference in the melatonin group compared to the control group (P = 0.03). However, after adjusting for baseline values of IL-6, no significant change was detected between the study groups following melatonin supplementation (*p* > 0.05) (Table 4).

**Table 4:** Oxidative stress and inflammatory markers of the study subjects at baseline and after the 10-week intervention

Parameter	Time	Groups		Mean difference	95% CI		*P* ^a^
		Control (*n* = 21)	Melatonin (*n* = 20)		Lower	Upper	
Hs CRP	Before	4810.3 ± 3047.2	4219.2 ± 3231.9	591.05	-1392.5	2574.6	0.55^**†**^
	After	4331.6 ± 2828.6	3023.1 ± 1964	1189.3	-165.7	2544.3	0.12 ^**ƪ**^
	Change	−520.5 ± 2059	-1578 ± 2976	1057.9	-722.9	2838	0.23^**†**^
TOS	Before	5.89 (5.33 – 6.40)	5.82 (5.29 – 6.61)	−0.33	-1.51	0.84	0.92^**‡**^
	After	6.23 (5.59 – 6.59)	5.8 (4.99 – 6.59)	0.97	-0.02	2.2	0.22 ^**ƪ**^
	Change	0.56 (−0.25 – 1.68)	-0.43 (-1.23 – 0.74)	1.07	-0.46	2.62	0.13^**‡**^
IL-6	Before	57.27 (53.45 – 59.99)	56.72 (54.54 – 59.99)	0.81	-5.41	7.05	1.0^**‡**^
	After	62.17 (59.99 – 65.43)	56.72 (55.63 – 59.99)	11.7	-6.9	30.4	0.4 ^**ƪ**^
	Change	7.63 (2.18 – 17.43)	-0.54 (−3.27 – 3.27)	12.88	-6.4	32.16	0.03^**‡**^
TAC	Before	0.19 (0.19 – 0.21)	0.2 (0.19 – 0.22)	−0.002	-0.017	0.012	0.71^**‡**^
	After	0.2 (0.19 – 0.25)	0.19 (0.18 – 0.2)	0.003	-0.01	0.02	0.78 ^**ƪ**^
	Change	0 (−0.02 – 0.01)	-0.01 (−0.03 – 0.01)	0.003	-0.022	0.029	0.68^**‡**^
MDA	Before	12.8 ± 2.8	14.3 ± 8	−1.58	1.85	-5.41	0.4^**†**^
	After	12.3 ± 2.9	14.1 ± 5.3	−1.9	-4.9	1.05	0.22 ^**ƪ**^
	Change	−0.54 ± 3.94	-0.98 ± 10.8	0.44	-5.3	6.27	0.87^**†**^

*Hs CRP* high-sensitivity C-reactive protein, *TOS* total oxidative stress, *IL-6* interleukin-6, *TAC* total antioxidant capacity, *MDA* malondialdehyde

Data are presented as mean ± standard deviation or Median (IQR)

^a^ based on ^**†**^independent sample t-test, ^**‡**^ Mann–Whitney U-Test, and ^**ƪ**^ general linear model

## Discussion

With the increasing prevalence of chronic kidney disease (CKD) and its detrimental effects on individuals' quality of life, efforts are being made to identify efficient treatments and preventive measures. Several studies have explored the impact of various nutrients and supplements on inflammatory and oxidative levels in CKD patients, but the findings are still inconclusive. To the best of our knowledge, despite melatonin's pharmacological impact on oxidative stress and inflammatory markers, it has not been adequately evaluated in the diabetic CKD population. Moreover, many studies often do not delineate between diabetic patients with and without kidney disease. The unique pathophysiological characteristics of DKD and the complex interplay of multiple biological factors, limits the extrapolation of previous findings to the DKD population. The present randomized, double-blind, placebo-controlled clinical trial was carried out to address this gap by focusing exclusively on patients with DKD, providing more accurate data on the effects of melatonin supplementation on oxidative stress and inflammatory biomarkers, including TAC, TOS, and MDA for oxidative stress, as well as IL-6 and hs-CRP for inflammatory markers.

The obtained results of this clinical trial indicated that melatonin supplementation for 10 weeks did not improve markers of inflammation and oxidative stress including hs-CRP, IL-6, TOS, TAS, and MDA in diabetic patients with CKD. Also, anthropometric measures such as weight, BMI, and WC were not changed following 10-week melatonin supplementation in these patients.

Regarding studies, one clinical trial study demonstrated that three months of melatonin supplementation (3 mg/day) improved the lipid profile. However, melatonin did not significantly reduce inflammatory markers such as CRP [27].

In contrast to our findings, Ostadmohammadi et al. reported that 12 weeks of melatonin supplementation (2 × 5mg/day) in 30 diabetic hemodialysis patients led to favorable improvements in hs-CRP, TAC, and MDA, among other inflammatory and oxidative markers. However, they did not evaluate the effect of melatonin administration on other biomarkers of oxidative stress and inflammatory factors such as IL-6 [28]. Consistent with these result0s, melatonin administration has been shown to improve antioxidant defenses and alleviate chronic inflammation in diabetic patients through a systematic review and meta-analysis of 14 trials. Significant decreases in CRP, TNF-α, IL-1, IL-6, and MDA levels were seen, accompanied by increases in TAC, GSH, and SOD [29]. Cutando et al. [30] also found that six weeks of melatonin administration significantly decreased serum hsCRP levels in patients with non-alcoholic hepatic steatosis, though other inflammatory and oxidative markers were not examined in that study. Discrepancies between our findings and those of previous studies may be attributed to differences in study design, melatonin dosage, follow-up periods, different sample sizes, study population, and use of various methods to measure inflammatory and oxidative stress biomarkers.

Several mechanisms may explain melatonin’s effect on oxidative stress and inflammatory markers in diabetic patients with CKD. Melatonin reduces MDA levels by acting as a direct free radical scavenger and an antioxidant. It detoxifies harmful radicals such as hydroxyl radicals, peroxynitrite, nitric oxide, and singlet oxygen, which contribute to lipid peroxidation and MDA elevation. By neutralizing these radicals, melatonin helps lower MDA levels [31–38]. An end-product of lipid peroxidation that serves as a marker of oxidative stress in CKD [39]. Melatonin supplementation has also been shown to significantly increases TAC, likely due to its ability to enhance antioxidant levels such as GSH and boost the activity of antioxidant enzymes like CAT, SOD, GPx, and GR. These factors contribute to TAC levels in human plasma. Melatonin also activates transcriptional regulation factors, through its receptors (MT1 and MT2), enhancing the oxidant defense system, and reducing ROS accumulation [40–46], which parallels the finding that habitual intake of the dietary TAC is associated with a lower risk of CKD [47]. Additionally, melatonin might inhibit prostaglandins production, the synthesis of adhesion molecules [48, 49], and cyclooxygenase-2 expression in macrophages [50–52] along with decreasing the polymorphonuclear cell accumulation at inflammatory site [49, 53]. Excessive reactive oxygen production also plays a significant role in inflammation [54, 55], and melatonin's antioxidant properties enable it to scavenge free radicals and activate endogenous antioxidant defenses, thus preventing inflammatory processes [56, 57]. Furthermore, melatonin also acts on T cells and monocytes, increasing IL-2 and IL-6 production, respectively. Cultured monocytes respond to melatonin by increasing IL-6 production, and melatonin has been shown to stimulate IL-1, IL-6 and TNF production by isolated monocytes [58]. Interleukin-6 accelerates the progression of CKD by aggravating kidney injury and initiating its complications, especially the chronic vascular disease (CVD) [59]. Moreover, melatonin may reduce inflammation through downregulation nuclear factor kappa B [60], which suppresses pro-inflammatory markers like hs-CRP [55] Additionally, melatonin may decrease inflammatory markers by scavenging toxic oxygen derivatives in inflamed tissues [61].

### Strengths and limitations of the study

One of this research's most significant advantages was that we estimated the inflammatory markers like IL-6 and hs-CRP. Furthermore, this is the first investigation into the effects of melatonin supplementation on the oxidative and inflammatory profiles of CKD patients with diabetes. However, there are a few restrictions to be aware of, though. As one of the most important limitations, initially, the trial's sample size was estimated to include 24 people in the control and intervention groups; however, three persons withdrew unexpectedly. Second, the melatonin serum concentration was not determined in this investigation. Third, higher dosages of melatonin supplements are an option. Finally, a longer trial duration may have improved our ability to evaluate the effect of melatonin on oxidative and inflammatory markers in CKD patients with diabetes.

## Conclusion

Overall, our findings showed that melatonin supplementation did not significantly change oxidative stress levels and inflammatory indicators, including TAC, TOS, MDA, IL-6, and hs-CRP in blood samples in diabetic patients with CKD. IL-6, a measure of inflammation, changed significantly after 10 weeks of melatonin administration; however, this effect was not statistically significant when we took baseline levels into account. We suggested that more randomized controlled trials should be conducted with larger sample sizes, higher melatonin dosages, and longer follow-up times to more thoroughly evaluate how melatonin affects inflammatory markers and oxidative stress in diabetic patients with CKD.

### Trial status

The registration date was 11 August 2023 (protocol version: 1.0). The recruitment started on 20 October 2023 and almost finished on 5 May 2024.

## Data Availability

The first and corresponding authors had access to interim results and decided to terminate the trial. The non-identifiable individual patients’ data became available to other researchers in academic institutions. The datasets analyzed during the current study and statistical code are available from the corresponding author on reasonable request, as is the full protocol.

## Abbreviations

BMI: Body mass index
BP: Blood pressure
CKD: Chronic kidney disease
DKD: Diabetic kidney disease
DM: Diabetes mellitus
ELISA: Enzyme-linked immunoassay
ESRD: End-stage renal disease
GFR: Glomerular filtration rate
GR: Glutathione Reductase
GSH: γ-l-glutamyl-l-cysteinyl-glycine
HTN: Hypertension
hs-CRP: High-sensitivity C-reactive protein
IL: Interleukin
IPAQ-SF: International physical activity questionnaire – short form
IRCT: Iranian registry of clinical trials
MDA: Malondialdehyde
MET: Metabolic equivalent
MT: Melatonin receptor
NSAIDs: Non-steroidal anti-inflammatory drugs
PKD: Polycystic kidney disease
SD: Standard deviation
SOD: Superoxide dismutase
TAC: Total antioxidant capacity
TOS: Total oxidative stress
TNF: Tumour-necrosis factor
WC: Waist circumference
